# Cloning, Expression and Functional Characterization of In-House Prepared Human Basic Fibroblast Growth Factor

**Published:** 2013-02-20

**Authors:** Hassan Rassouli, Mohammad Sharif Tabe Bordbar, Mehran Rezaei Larijani, Mohammad Pakzad, Hossein Baharvand, Ghasem Hosseini Salekdeh

**Affiliations:** 1. Department of Molecular Systems Biology at Cell Science Research Center, Royan Institute for Stem Cell Biology and Technology, ACECR, Tehran, Iran; 2. Department of Stem Cells and Developmental Biology at Cell Science Research Center, Royan Institute for Stem Cell Biology and Technology, ACECR, Tehran, Iran; 3. Department of Developmental Biology, University of Science and Culture, ACECR, Tehran, Iran

**Keywords:** Basic Fibroblast Growth Factor (bFGF), Recombinant Protein, Embryonic Stem Cells, Cell Proliferation, Pluripotency

## Abstract

**Objective:**

Human basic fibroblast growth factor (bFGF) plays an important role in cellular proliferation, embryonic development, and angiogenesis as well as in several signaling pathways of various cell types. bFGF is an essential growth factor for the maintenance of undifferentiated human embryonic stem cells (hESCs) and human induced pluripotent stem cells (hiPSCs).

**Materials and Methods:**

In this experimental study, we present a straightforward method to produce biologically active recombinant human bFGF protein in E. coli that has long-term storage ability.

**Results:**

This procedure provides a rapid, cost effective purification of a soluble human bFGF protein that is biologically active and functional as measured in hESCs and hiPSCs *in vitro* and *in vivo*.

**Conclusion:**

The results show no significant difference in function between our in-house produced and commercialized bFGF.

## Introduction

Human embryonic stem cells (hESCs) and human induced pluripotent stem cells (hiPSCs) have the unique ability to differentiate into all cell types of the three germ layers. They also have an unlimited ability for proliferation that can be employed *in vitro* to generate the desired cells for cell therapies and developmental biology studies (1).

These cells are grown in complex culture mediums that contain several growth factors and cytokines (2). One essential growth factor for the maintenance of hESCs and hiPSCs in a pluripotent and undifferentiated state is basic fibroblast growth factor (bFGF or FGF-2) (3). bFGF, a member of the fibroblast growth factor (FGF) family, is known to play a role in the proliferation and differentiation of certain cell types, such as hESCs, hiPSCs (4), and neural progenitors (5, 6). This growth factor also shows potent angiogenic effects *in vivo* and *in vitro*, stimulates the growth of smooth muscle cells, and assists in wound healing and tissue regeneration (7). To date, approximately 24 FGF members and 4 FGF receptors (FGFR) have been identified (8).

Numerous reports have discussed the successful production of growth factors and cytokines such as interleukin-1 (9), interleukin-4 (10), epidermal growth factor (11, 12), FGF-2 (13, 14), and leukemia inhibitory factor (15-17) in E. coli for both research and clinical purposes. There are also reports that discuss an increased recombinant bFGF yield by using codon optimization (18). Although there are reports regarding the production of recombinant human bFGF, additional steps of downstream processing are necessary for the improvement of its functionality and long-term storage. Here we describe a straightforward strategy to produce biologically active recombinant human bFGF protein in E. coli that has long-term storage capability. This procedure provides both a rapid and cost effective purification of soluble human bFGF protein that is biologically active and functional. This protocol could additionally be used for the production of other growth factors.

## Materials and Methods

### Cloning of the C-terminal fragment of bFGF cDNA

In this experimental study, total RNA was isolated using NucleoSpin RNA II (MNCo; Germany) from human fibroblast cells. After isolation, total RNA was treated with RNase-free DNase (Invitrogen, Carlsbad, CA, USA) to ensure the complete removal of genomic DNA. The first strand of cDNA synthesis was performed using Super Script III reverse transcriptase (Invitrogen, Carlsbad, CA, USA), oligo dT primer, and 2 µg of purified total RNA. The primers used to amplify bFGF were designed from Genbank (Accession No. NM_002006.4) nucleotides 429-864 to exclude the N-terminal propeptide. The C-terminal fragment of bFGF was amplified with pET-bFGF-f) 5' AAT TAA GAA TTC ATG GCA GCC GGG AGC ATC 3') and pET-bFGF-r (5' TAC CAT GAG CTC TCA ACT CTT AGC AGA CAT TGG 3').

These primers introduced an EcoRI restriction site at the 5' end and a SacI restriction site at the 3' end of the amplicon. For fragment amplification, pfx DNA polymerase (Invitrogen, Carlsbad, CA, USA) and a Mastercycler® Gradient PCR (Eppendorf Netheler-Hinz GmbH, Hamburg, Germany) were used. Amplification steps included: pre-incubation at 95℃ for 4 minutes; 30 cycles at 95℃ for 30 seconds, 60℃ for 30 seconds, and 68℃ for 40 seconds; followed by one incubation step at 68℃ for 8 minutes. Next, the PCR products were analyzed by electrophoresis on 1.5% agarose gel and visualized by ethidium bromide staining under ultra violet (UV) light.

### Construction of pET28/bFGF expression vector

The PCR product was digested with EcoRI and SacI restriction enzymes (Roche Applied Science, Basel, Switzerland), cloned in a pET 28a vector and digested with the same restriction enzymes. Expression of His-tag fused bFGF, cloned in pET 28, was under the direct control of the T7 promoter and transcription terminator. The recombinant expression vector construct that carried the bFGF gene (pET 28/bFGF) was transferred into E. coli strain BL 21 (DE 3) competent cells (Novagen, Madison, WI, USA) by the heat shock method as described by the manufacturer (User Protocol TB 009 Rev. F 0104). The transgene nucleotide sequence in pET 28/bFGF was analyzed by DNA sequencing.

### Recombinant fusion protein expression and purification

For recombinant protein expression, clones with the correct sequence were grown overnight in LB medium that contained 50 mg/ml kanamycin at 37℃ and shaken at 180 rpm. Next, cultures were diluted 1:100 in fresh LB that contained 50 mg/ml kanamycin and 2% glucose, and then cultivated at 37℃ until the OD_600_ of the media reached 0.8. Recombinant fusion protein expression was then induced by the addition of isopropyl-d-thiogalactopyranoside (IPTG; Fermentas, Lithuania). In order to optimize the highest yield of soluble recombinant fusion proteins, different IPTG concentrations (0.2, 0.5, or 1.0 mM) and induction temperatures (30℃ or 37℃) were used and cells were grown for 6 hours or longer. Induced cells were harvested by centrifugation at 8000×g for 10 minutes; cell pellets were then frozen at −80℃ until use for protein purification.

Prior to purification, cell pellets were thawed and resuspended in 10 ml lysis/binding buffer/g of cells, and incubated on ice for 30 minutes. The lysate was further disrupted by sonication on ice for 10 minutes with 45 second pulses and a 15 second rest period between pulses. The cell debris was sedimented by centrifugation at 14000×g for 30 minutes and the supernatant used for purification. Recombinant His6-bFGF fusion protein was purified by the Ni-NTA Fast Start Kit (Qiagen, USA). The column was washed with 10 ml of washing buffer [20 mM Tris–HCl (pH= 8.0), 150 mM NaCl, and 25 mM imidazole] to remove non-specifically bound proteins. His-tag fused recombinant proteins that remained on the column were eluted with 1 ml elution buffer that contained 250 mM imidazole in three separate fractions. In each step, 20 µl samples were preserved for further analysis by SDS-PAGE.

The concentration of the purified protein was determined by the Bradford method. The purified His6-bFGF fusion protein (Royan-bFGF) was dissolved in storage buffer, filter sterilized (0.2 µm), distributed into vials (100 µg per vial), lyophilized, and stored at -80℃. In order to determine the best storage buffer we used three groups: i. a group that contained Tween 20; ii. a group that contained β-mercaptoethanol; and iii. a group that contained both Tween 20 and β-mercaptoethanol. In addition, there were two groups in which the first group contained bacteria treated with sonication in the purification stages. The second group was not treated by sonication.

### SDS-PAGE and mass spectrometry analysis

Identical volumes of different elution fractions were mixed with a 1/5 volume of 5× loading buffer [1 M Tris-HCl (pH= 6.8), 10% w/v SDS, 0.05% w/v bromophenol blue, 50% glycerol, and 200 mM β-mercaptoethanol] and heated at 95℃ for 5 minutes prior to SDS-PAGE using a 12% (w/v) separating gel followed by staining with 0.1% Coomassie brilliant blue R-250. Bands of interest were excised from the SDS-PAGE gel and samples analyzed by liquid chromatography coupled with tandem mass spectrometry (LC-MS/MS) at York University.

### Functional assay of Royan-bFGF

The cells lines were expanded as previously described in feeder-free conditions (19, 20) to culture and passage hESC (Royan H5 and Royan H6) (21) and hiPSC (R1-hiPSC1 and R1-hiPSC4) lines (22). We used Royan-bFGF along with commercial bFGF obtained from Sigma and Invitrogen with the intent to compare their functionality and efficiency. The cell lines were treated with three different concentrations of Royan-bFGF (100, 200 and 300 ng/ ml) and a 100 ng/ml concentration of commercial bFGFs and there was a negative control in all these steps. Efficacies of different recombinant proteins were compared during four passages. Morphology, differentiation status, and multiplication rate were the criteria used for comparison. For the past three years, RoyanbFGF has been routinely used in Royan Institute’s Cell Culture laboratories. To ensure the proper function of Royan-bFGF, we analyzed the karyotype and some typical stem cell markers of the cell lines after several passages.

### Culturing hESCs and hiPSCs

The cells were cultured in hESC medium that contained DMEM/F12 (Invitrogen, 21331-020) supplemented with 20% knockout serum replacement (KOSR, Invitrogen, 10828-028), 2 mM L-glutamine (Invitrogen, 25030-024), 0.1 mM β-mercaptoethanol (Sigma-Aldrich, M7522), 1% nonessential amino acids (Invitrogen, 11140-035), 100 units/ml penicillin, 100 µg/ml streptomycin (Invitrogen, 15070-063), insulin-transferrin-selenite (ITS, Invitrogen, 41400- 045), and 100 ng/ml bFGF (Sigma-Aldrich, F0291, Invitrogen, PHG0021L, and Royan). Cells were grown in 5% CO_2_ at 95% humidity and passaged every seven days. For passaging, cells were washed once with Dulbecco’s phosphate-buffered saline that contained Ca^2+^ and Mg^2+^ (DPBS, Invitrogen, 14040- 117) and then incubated with DMEM/F12 that contained 1:1 collagenase IV (0.5 mg/ml, Invitrogen, 17104-019):dispase (1 mg/ml, Invitrogen, 17105- 041) at 37℃ for 5-7 minutes. When colonies at the edge of the dish began to dissociate from the bottom, the enzyme was removed and colonies were washed with DPBS. Cells were collected by gentle pipetting and replated on Matrigel (0.3 mg/ml, Sigma-Aldrich, E1270)-coated dishes. The medium was changed every other day.

### Teratocarcinoma formation

Colonies with undifferentiated morphologies were collected by trypsin/EDTA (1x, Invitrogen, 25300-054) treatment, dissolved in 50 µL Matrigel, and then approximately 2×10^6^ cells were injected into testis of six-week old Nude mice. Teratomas that developed around ten weeks after injection were surgically removed and fixed in Bouin’s Fixative for four days at room temperature (RT) and subsequently embedded in paraffin. Samples were sectioned at a thickness of 6 µM, processed with hematoxylin and eosin staining, and observed under a bright field microscope.

### Karyotype analysis

Karyotype analysis was performed as explained by Mollamohammadi et al. (21). Cells were treated with thymidine (0.62 mM, Sigma-Aldrich, T1895) for 16 hours at 37℃ in 5% CO_2_ and then washed. Next, cells were left for 5 hours and then treated with 0.15 mg/ml colcemid (Invitrogen, 15210- 040) for 30 minutes. Cultured hiPSCs and hESCs were exposed to 0.075 M KCl at 37℃ for 16 minutes, after which cells were fixed in three consecutive immersions in ice-cold methanol/glacial acetic acid (3:1) and subsequently dropped onto pre-cleaned, chilled slides. Chromosomes were visualized using standard G-band staining. At least 20 metaphase spreads were screened and 10 were evaluated for chromosomal re-arrangements.

### Immunofluorescence and alkaline phosphatase staining

Cells were fixed in 4% paraformaldehyde for 20 minutes, then permeabilized with 0.2% Triton X-100 for 30 minutes and blocked in 10% goat serum in PBS for 60 minutes. Primary antibody incubation was performed for 1 hour at 37℃, washed 3 times, and incubated with FITC-conjugated secondary antibodies, anti-mouse IgM (1:100, Sigma-Aldrich, F9259), anti-rat IgM (1:200, eBioscience, 11-0990), and anti-mouse IgG (1:200, Sigma-Aldrich, F9006) as appropriate for 1 hour at 37℃. Primary antibodies used to determine the undifferentiated states of hESCs and hiPSCs were: anti-TRA-1-60 (1:100, Chemicon MAB4360), anti- TRA-1-81 (1:100, Chemicon MAB4381), anti-Oct-4 (1:100, Santa Cruz Biotechnology, SC-5279), anti- SSEA-3 (1:50, Chemicon, MAB4303), and anti- Nanog (1:100, Sigma-Aldrich, N3038). For nuclei staining we used DAPI (0.1 µg/ml, Sigma-Aldrich, D8417). A fluorescent microscope (Olympus, Japan) was used for cell analysis. Alkaline phosphatase staining was performed according to the manufacturer’s recommendations (Sigma-Aldrich, 86R, USA).

### Comparison of freshly prepared and lyophilized Royan-bFGF

One of the most important issues in recombinant protein production is its long-term storage. Repeated freezing and thawing may interfere with recombinant protein structure and function. Lyophilizing is the best choice for long-term maintenance, although some proteins may lose their function during this process.

To investigate the efficacy of freeze-dried and freshly produced Royan-bFGF, we used cell culture assays to compare freshly prepared and lyophilized recombinant proteins. Fresh and lyophilized Royan-bFGF (300 ng/ml) were compared with commercial bFGFs from Sigma-Aldrich and Invitrogen (100 ng/ml). Royan H6 and R1-hiPSC4 were treated in five groups: four groups cultured by the above mentioned bFGFs and the fifth group that was not treated with any bFGF. This experiment was performed for four passages and then scored.

### Royan-bFGF stability assay

We evaluated the long-term stability of fresh and freeze-dried bFGFs. The functionality of freshlyprepared Royan-bFGF at 2, 4, 6, and 8 months (maintained at 4℃) after production and lyophilized Royan-bFGF (6, 12, 18, 24, and 36 monthold) recombinant proteins were assessed. Freezedried proteins (kept at -80℃) were evaluated as described above. All samples were dissolved in sterile tris buffer (5 mM, MP Biomedicals, 816100) and stored at 4℃ prior to analysis.

### Endotoxin testing

Endotoxin test was performed with a PYROGENT ™ Gel Clot LAL Assay Kit (Lonza, Basel, Switzerland) according to the manufacturer’s instructions. This method is based on the fact that gram-negative bacterial endotoxin catalyzes the activation of a proenzyme in limulus amebocyte lysate (LAL). The initial rate of activation is determined by the concentration of endotoxin that is present. The activated enzyme (coagulase) hydrolyzes specific bonds within a clotting protein (coagulogen) that is also present in the LAL. Once hydrolyzed, the resultant coagulin self-associates and forms a gelatinous clot. The turbidimetric LAL assay measures the increase in turbidity (optical density) that precedes the formation of the gel clot.

LAL is manufactured from horseshoe crab blood cells. These cells are isolated from the serum by centrifugation and then placed in distilled water, which causes them to swell and burst or lyse. This process results in the release of chemicals from the inside of the cell, which is termed the "lysate". The lysate is subsequently purified and freeze-dried. To test a sample for endotoxins, it is mixed with lysate and water. Three samples from each production series were utilized for the endotoxin detection assay.

## Results

### Construction of bFGF fusion protein expression vector

**Fig 1:**
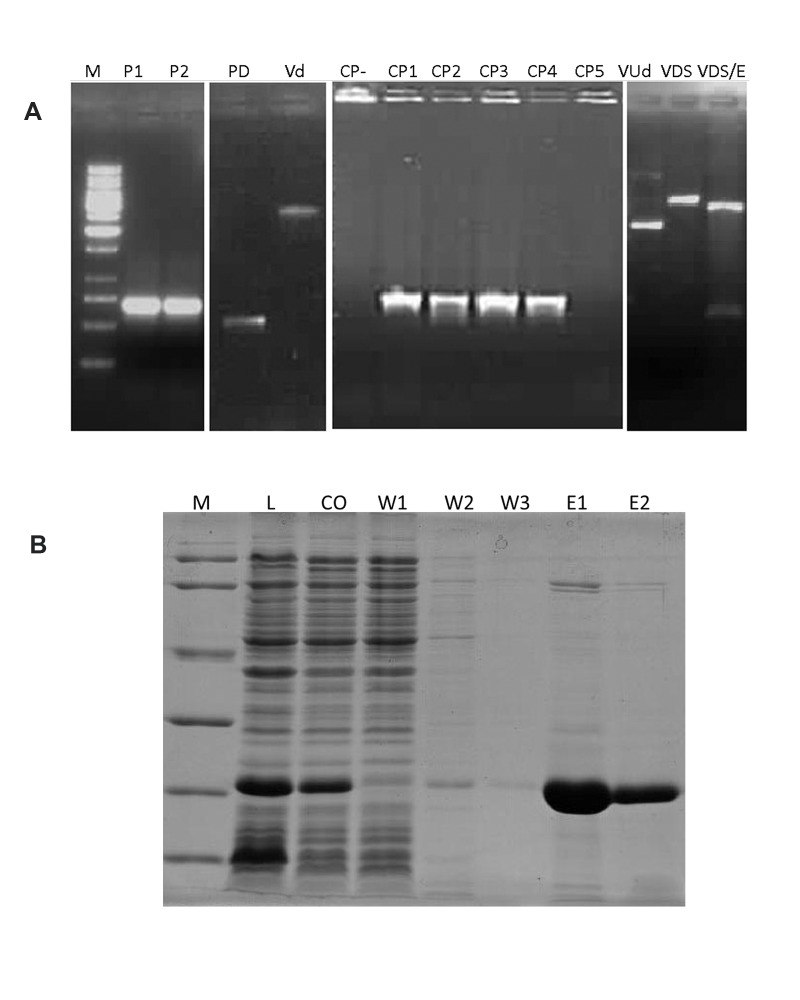
PCR from amplified bFGF and SDS-PAGE of produced bFGF. A. The expected 459 bp product of bFGF amplified by PCR with primers that added restriction sites at both ends. M; Size marker, P1 and P2 bFGF PCR product bands, PD; bFGF PCR product digested with SacI/EcoRI, VD; pET28 vector digested with SacI/EcoRI, CP-; Colony PCR negative control, CP1-5; Colony PCR results for clones 1 to 5, VUd; Undigested pET28/bFGF vector, VDS; pET28/ bFGF vector digested with SacI, VDS/E and pET28/bFGF vector digested with SacI /EcoRI. B. SDS-PAGE results for different steps of bFGF production. Recombinant his-tagbFGF expressed and purified at a good concentration and purity. M; Protein size marker, L: Lysate, CO; Cut off, W1; Wash 1, W2; Wash 2, W3; Wash 3, E1; Elution 1 and E2; Elution 2.

The 435 bp bFGF was amplified from human fibroblast cell mRNA and subsequently cloned in a pET28 expression vector to produce the N-terminus His6-bFGF recombinant in E. coli BL21 (DE3; Fig 1). We used the native sequence of bFGF with no modifications, such as the replacement of GC-rich regions with ATrich regions or the replacement of rare codons. The pET28/bFGF construct was confirmed by a PCR that utilized T7 forward and bFGF reverse primers. This PCR showed the bFGF insertion in the vector, as well as the correct orientation critical for protein production.

### Expression and purification of recombinant bFGF fusion protein

The pET28/bFGF that encoded the His6-bFGF fusion protein was transferred into E. coli BL21 (DE3). As mentioned above, different IPTG concentrations (0.2, 0.5 or 1.0 mM) and induction temperatures (30℃ or 37℃) were used. Expression of the His6- bFGF fusion protein after 6 hours of induction yielded more fusion proteins at the 37℃ induction temperature compared to the 30℃ temperature. However, at 37℃ there were inclusion bodies observed. Thus we decided to use 30℃ as the induction temperature. We determined the best concentration of IPTG to be 0.2 mM with regards to expression level and inclusion body formation. Over-expressed recombinant bFGF were purified by the Ni-NTA Fast Start Kit and the target protein was eluted by 250 mM imidazole. Most contaminant bacterial proteins were eliminated in the flow through and washing steps. None of the specifically bound proteins were further reduced by washing the resin or by increasing concentrations of imidazole. Finally, most of the binding impurities were eluted at 25 mM imidazole. The bFGF recombinant protein (approximately 17 kDa) was obtained in the 250 mM imidazole fractions (Fig 1A). The identity of the purified bFGF fusion proteins was confirmed by mass spectrometry analysis of the protein band excised from the SDS-PAGE gel (Fig 1B). The results indicated that our fusion proteins matched bFGF (GenBank Accession No. NM_002006.4).

### Analysis of the effects of produced bFGF on hESC and hiPSC pluripotency and self-renewal

**Fig 2:**
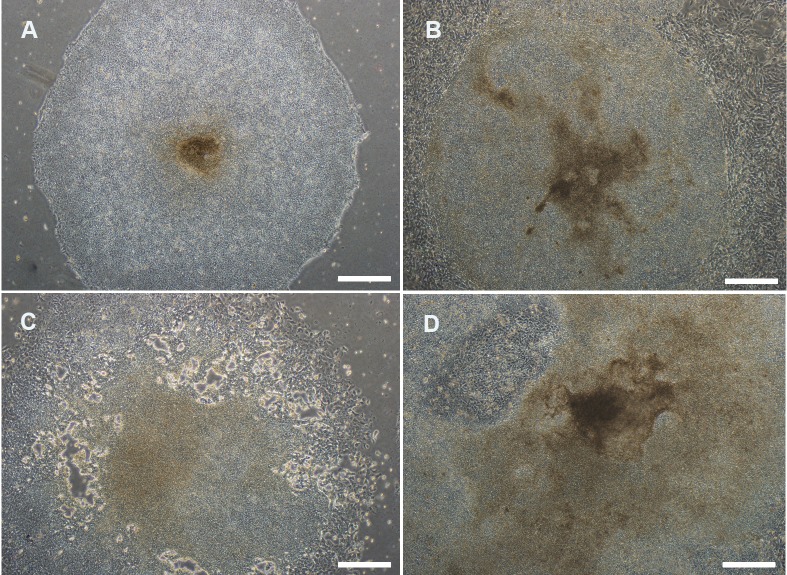
Evaluation of human pluripotent stem cell colonies based on morphology seven days post-subculture (grades A-D). The quality of colonies was graded as follows: Grade A. (excellent) colonies had even morphology and well-defined edges. The cells in the colonies were dense and individual cells could not be easily distinguished. The colonies were thick, multilayered, homogenous, and exhibited 0-30% differentiation. The differentiated cells migrated and passed from the periphery of the colonies. Grade B. (good) with differentiation in 30-50% of the peripheral area. Grade C. (fair) colonies exhibited more than 50-80% differentiation. Grade D. (poor) colonies differentiated more than 80-100%, and exhibited a well differentiated morphology with inhomogeneous levels.

**Fig 3:**
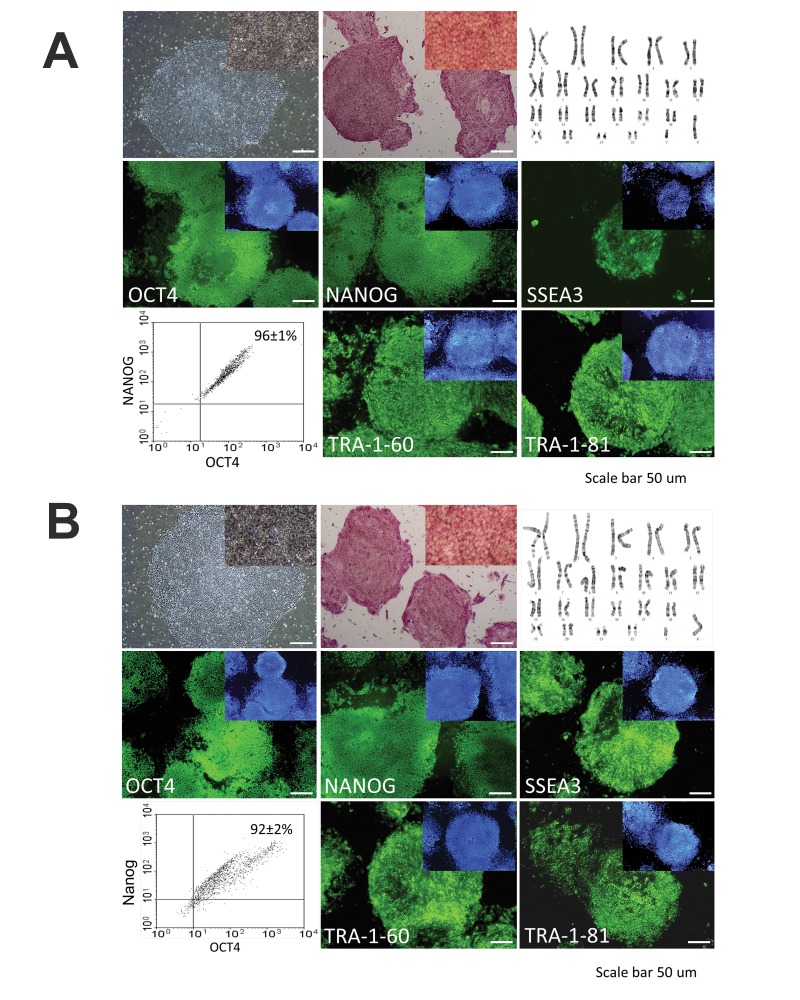
Characterization of human embryonic (hESCs) and induced pluripotent stem cells (hiPSCs) expanded in the presence of bFGF. Lines are characterized after 15 to 18 passages. The cell lines retained key properties of pluripotent markers. Morphology of human embryonic (hESCs) and induced pluripotent stem cells (hiPSCs) and expressions of ALP, Oct4, Nanog, SSEA3, TRA-1-60, and TRA-1-81. Nuclei were stained with DAPI (blue). Dot plot diagram of flow cytometry shows co-expression of two markers. The karyotype of hESCs and hiPSCs after several passages with Royan-bFGF was normal.

All three samples of each bFGF production batch used for the endotoxin detection assay were endotoxin-free. To investigate our bFGF product functionality, we applied it to the culture and passaging of our cell lines, Royan H6 and R1-hiPSC4. We divided our experiment into different groups that were treated with 100, 200, and 300 ng/ml concentrations of Royan bFGF, which was compared to Sigma- Aldrich and Invitrogen bFGFs. The results showed that the group treated with 300 ng/ml of Royan bFGF was similar to the groups treated with bFGF from both companies in terms of their morphological characteristics (Fig 2, Table 1). Royan bFGF administered for four subsequent passages of our cell lines showed undifferentiated morphology when compared with the control groups. After 18 passages for Royan H6 and 15 passages for R1-hiPSC4, the treated cells preserved their normal karyotypes. After immunostaining for pluripotency markers, these cells stained positive for Oct4, Nanog, SSEA3, TRA-1-60, and TRA-1-81 (Fig 3). We repeated this assay with our other human ES cell lines, Royan H5 and R1-hiPSC1; all lines displayed similar results (data not shown).

**Table 1:** Comparison of Royan bFGF with commercial bFGFs


Treatment	Percent of human embryonic stem cell (hESC) grade A+B colonies.				
	P1	P2	P3	P4	Average

NTL	60(1:3)	70(1:2)	70(1:3)	65	<0.05
SML	60(1:3)	70(1:2)	60(1:3)	60	
STML	70(1:4)	70(1:4)	70(1:4)	70	0.028
STMF	80(1:4)	80(1:4)	70(1:4)	75	
Sigma bFGF	70(1:4)	70(1:4)	70(1:4)	70	0.566
Invitrogen bFGF	70(1:4)	60(1:4)	70(1:4)	65	


All groups contain some treatment that the each treatment is shown in a capital letter S; Sonicated, N; Not sonicated, T; Tween 20, M: β-mercaptoethanol, L; Lyophilized and F; Fresh bFGF. NTL; Not treated with sonicator in the purification process. NTL contains Tween 20 in storage buffer and was lyophilized for long-term storage. STMF group: Treated with sonication, contained Tween 20 and β-mercaptoethanol in storage buffer and maintained at 4℃ without lyophilization. The percent of colonies that were grades A or B (A+B, see Fig 2) during each passage, P1-4: Passages 1-4, (1:n): Indicates proliferation rate during each passage. Clearly, the STMF group is the best group because it has highest percentage of undifferentiated cells and proliferation rate.

### Comparison of fresh and lyophilized bFGF and their stability assays

No significant difference was observed between fresh and lyophilized bFGF as evaluated by the cell culture test (Table 1). These results indicated that bFGFs maintained their structure during the freeze-drying process. We also did not observe any significant reduction in the functionality of lyophilized bFGF even after 36 months. However, the function of nonlyophilized Royan-bFGFs decreased substantially after six months when compared with freshly prepared Royan-bFGFs.

**Fig 4:**
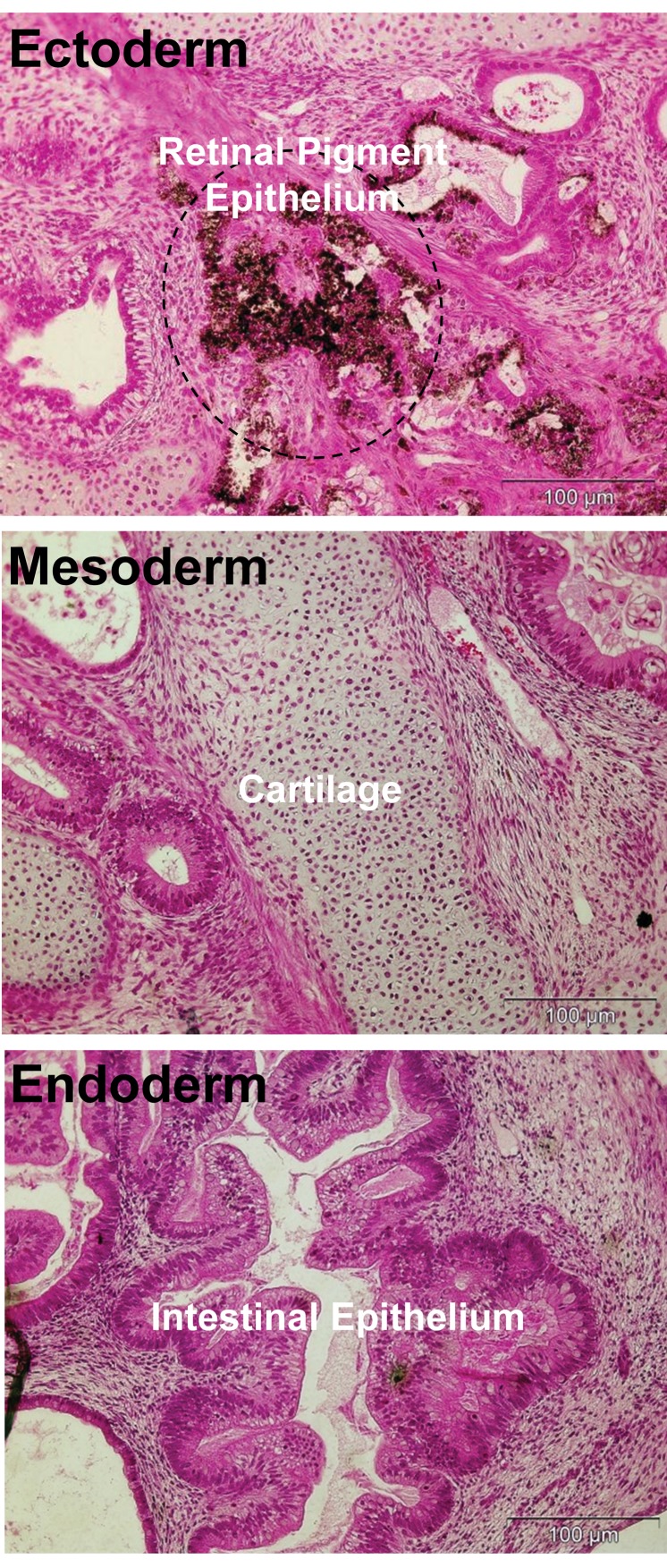
Teratoma formation by hESC line in the presence of Royan-bFGF for more than six month continues continuous culture. Hematoxylin and eosin staining of paraffin sections of teratomas represent the differentiation of Royan H6 cells into various tissues, including retinal pigment epithelium as a remarkable tissue of ectoderm, cartilage (mesoderm), and intestinal epithelium (endoderm).

## Discussion

In the present study we isolated the cDNA encoding human bFGF cloned in pET28 vector. The pET28 expression vector has frequently been employed to efficiently and effectively express recombinant proteins in prokaryotic cells.

This vector has an exceptionally strong promoter that allows for high-level production of recombinant proteins (23). This is consistent with our results, which has shown high expression of Royan-bFGF in pET28. We also observed high protein yield with correct folding and without any inclusion body, which suggested the cost-effectiveness of the Royan-bFGF production.

Endotoxins can cause serious problems in cell cultures and interfere with other treatments (24, 25). The endotoxin test result of bFGF has shown that all samples were endotoxin- free or their endotoxins were under the detectable range. This has implied that Royan-bFGF can be used without endotoxin concerns.

No significant difference was observed between Royan-bFGF and commercial bFGF as evaluated with hESCs and hiPSCs culture. Human iPSCs/ESCs cultured in the presence of Royan-bFGF could propagate with typical round and flat morphology with definite margins and a high nucleus-cytoplasm ratio, maintain genetic stability, express alkaline phosphatase (ALP) and the most important stemness factors, including Oct4 and Nanog, in addition to the surface markers TRA 1-60, TRA 1-81, and SSEA3.

## Conclusion

Our results indicate that there is not a significant difference in the function of in-house generated and commercialized bFGF. The availability of large quantities of recombinant bFGFs should greatly facilitate the culture of hESCs and hiPSCs. Future studies aimed at elucidating the biological function of FGFs should allow for extensive clinical testing of these proteins for potential pharmaceutical use.
